# Transcriptome Analysis of Three Sheep Intestinal Regions reveals Key Pathways and Hub Regulatory Genes of Large Intestinal Lipid Metabolism

**DOI:** 10.1038/s41598-017-05551-2

**Published:** 2017-07-13

**Authors:** Tianle Chao, Guizhi Wang, Zhibin Ji, Zhaohua Liu, Lei Hou, Jin Wang, Jianmin Wang

**Affiliations:** Shandong Provincial Key Laboratory of Animal Biotechnology and Disease Control and Prevention, College of Animal Science and Veterinary Medicine, Shandong Agricultural University, Taian, 271018 China

## Abstract

The large intestine, also known as the hindgut, is an important part of the animal digestive system. Recent studies on digestive system development in ruminants have focused on the rumen and the small intestine, but the molecular mechanisms underlying sheep large intestine metabolism remain poorly understood. To identify genes related to intestinal metabolism and to reveal molecular regulation mechanisms, we sequenced and compared the transcriptomes of mucosal epithelial tissues among the cecum, proximal colon and duodenum. A total of 4,221 transcripts from 3,254 genes were identified as differentially expressed transcripts. Between the large intestine and duodenum, differentially expressed transcripts were found to be significantly enriched in 6 metabolism-related pathways, among which PPAR signaling was identified as a key pathway. Three genes, *CPT1A*, *LPL* and *PCK1*, were identified as higher expression hub genes in the large intestine. Between the cecum and colon, differentially expressed transcripts were significantly enriched in 5 lipid metabolism related pathways, and *CEPT1* and *MBOAT1* were identified as hub genes. This study provides important information regarding the molecular mechanisms of intestinal metabolism in sheep and may provide a basis for further study.

## Introduction

Although the contribution of the large intestine to the total-tract nutrient digestion is substantially less than that of the rumen, large intestine metabolism affects animal production and health^1^. To date, experimental studies on large intestine metabolism in sheep have focused on the cecum and colon. The cecum and the proximal colon are the main fermentation regions of the sheep large intestine and are responsible for controlling the passage of chyme^2, 3^. The total amount of chyme in the large intestines of sheep ranges from 700 to 1,200 g, an amount is equivalent to 15 to 26% of the total rumen contents^4^. Several studies on sheep and cattle have reported that the retention time of chyme in the large intestine is shorter than that in the rumen^4–6^. However, the segment of the large intestine is narrow and long, and its absorption capacity per unit volume may exceed that of the other intestinal regions^7^.

Several studies have reported that in the rumen and large intestine, carbohydrates are fermented into volatile short-chain fatty acids (SCFAs) and gas at similar rates^8, 9^. The large intestine accounts for 8 to 17% of the total SCFAs absorbed from the digestive tract of ruminants^4, 8^, and these fatty acids supply 5% of the digestible energy intake^10^ and 10% of the total-tract gross energy usage in sheep^11^. Cecal chyme has been reported to have strong proteolysis, deaminase and urease activity. Indeed, the ratio of isobutyric acid to isovaleric acid in the cecum is often higher than that in the rumen^12, 13^. Additionally, diaminopimelate and muramic acid can be decomposed in the large intestine, whereas they remain undigested in the small intestine^14^. Overall, the large intestine plays an important role in the digestion and absorption of starch, cellulose, nitrogenous compounds and fatty acids (mainly SCFAs) in sheep, thus making its study important for understanding metabolic mechanisms at the transcriptomic level.

Recently, several studies have reported the successful transcriptome sequencing of multiple tissues in sheep brain^15^, uterus^16^, skeletal muscle^17–19^, mammary gland^20^, milk^21^, rumen^22^, and skin^23^ tissues. To the best of our knowledge, only two transcriptomic studies on sheep have recently been conducted on large intestine metabolism. Analyzing the reference sheep genome and transcriptomes from 40 different tissues, Jiang *et al*. have reported genes involved in lipid metabolism that exhibit increased and/or altered tissue expression patterns^24^. Using transcriptome data sequenced by Jiang *et al*.^24^, Xiang *et al*. have analyzed and reported gene expression profiles for the sheep gastrointestinal tract^25^. Both studies focused on rumen metabolism and analyzed large intestine tissues for comparison.

Accordingly, to improve understanding of the regulatory mechanisms of sheep large intestine metabolism, we performed a transcriptome sequencing analysis of intestinal mucosal epithelial tissues from small-tailed Han sheep (SH). Mucosal epithelial tissues from the cecum, proximal colon and duodenum were selected for RNA extraction and sequencing. In addition, the transcriptome data for three Texel sheep intestine tissues sequenced by Jiang *et al*.^24^ were also utilized to further dissect the details of gene expression in this study. This study significantly advances knowledge regarding sheep intestinal gene expression. Our study also provides new insights into the potential molecular mechanisms involved in regulation of sheep intestinal metabolism.

## Results

### Summary statistics for the sequencing data

In this research, pooled samples from three small-tailed Han sheep (SH) intestine regions (duodenum, cecum and colon) were sequenced. A total of 212,637,096 raw sequencing reads with a length of 100 bp were obtained from the transcriptome sequencing results of the three libraries. Among them, 1,577,572 reads were removed in the quality filtering step. A total of 211,059,524 clean reads were retained for mapping and transcriptome assembly. In addition, another 317,229,652 clean reads were obtained from the downloaded Texel sheep intestinal transcriptome sequencing data (ENA study accession PRJEB6169). As shown in Table 1, more than 70% of the clean reads mapped to the sheep reference genome (SH duodenum 71.50%, SH cecum 71.85%, SH colon 71.99%, Texel duodenum 70.65%, Texel cecum 70.89%, Texel colon 71.02%), and nearly 60% mapped to the reference genes (SH duodenum 58.74%, SH cecum 59.07%, SH colon 61.41%, Texel duodenum 58.17%, Texel cecum 58.35%, Texel colon 60.01%). After transcript assembling and gene locus classification, the reference gene mapped reads were annotated into 38,934 transcripts associated with 21,174 reference genes. Among them, 14,102 genes were detected with only one transcript expression, whereas the other 6,857 genes had two or more transcripts (up to 31).

**Table 1:** A summary of the sequencing read alignments to the sheep reference genome and genes.

Intestinal Region	Clean reads	Genome map Rate (%)	Gene map Rate (%)	Expressed Genes	Expressed Transcripts
SH Duodenum	71,473,808	71.50	58.74	19,237	30,612
SH Cecum	67,173,384	71.85	59.07	19,059	30,397
SH Colon	72,412,332	71.99	61.41	19,274	31,023
Texel Duodenum	96,591,908	70.65	58.17	19,290	31,517
Texel Cecum	149,878,848	70.89	58.35	19,585	32,988
Texel Colon	70,758,896	71.02	60.01	18,912	30,738

According to the FPKM values, the mapped transcripts were divided into four groups: an ultra-low expression group (<1 FPKM), a low expression group (≥1 FPKM to 10 FPKM), a moderate expression group (≥10 FPKM to 500 FPKM) and a high expression group (≥500 FPKM). More than one-quarter of these transcripts showed ultra-low expression in all 6 samples (<1 FPKM), whereas others showed expression ≥ 1 FPKM in at least one sample (Table 2). To ensure the accuracy of further analysis, an FPKM score of 1.0 in at least one sample was chosen as a minimum expression cutoff for the transcripts. At this setting, 27,591 transcripts from 16201 gene loci were subjected to differential expression analysis.

**Table 2:** Transcript expression profiles from three intestinal regions.

	SH Duodenum	SH Cecum	SH Colon	Texel Duodenum	Texel Cecum	Texel Colon
Highly expressed transcripts (≥500 FPKM)	171	180	161	173	174	202
Medium expressed transcripts (≥10 FPKM to 500 FPKM)	6,810	6,863	6,643	6,852	7,001	6,836
Lowly expressed transcripts (≥1 FPKM to 10 FPKM)	11,579	11,758	11,889	14,698	15,123	14,069
Ultra lowly expressed transcripts (<1 FPKM)	12,052	11,596	12,330	9,794	10,690	9,631

To check the batch effect between two biological replicates, we performed principal components analysis according to their transcript expression data. As shown in Supplementary Fig. 1A, colon samples were clustered as one group, while the other four samples were mixed into another group. To detect whether the data from two biological replicates are consistency on the transcript expression pattern level, the transcripts expression values were mean centered with Z-value, and a principal components analysis was performed with the Z-value data. As shown in Supplementary Fig. 1B, the 6 samples were divided into 3 groups according to their intestinal regions. This result suggests that performing analysis at the expression pattern level should be an effective way to reduce the impact caused by confounding variables. Therefore, differential expression analysis was performed on two biological replicates separately. The final differential expressed transcripts screening was performed by expression pattern analysis.

### Identification of Differentially Expressed Transcripts

To identify differentially expressed transcripts, a comparison among three intestine regions was separately performed in SH and Texel. To maximize the accuracy and reliability, overlapping differentially expressed transcripts from each of the two biological replicates were used for subsequent analyses. As a result, 4,221 transcripts from 3,254 gene loci were identified as differentially expressed transcripts (Supplementary Dataset 1). The number of differentially expressed transcripts identified in each specific comparison is shown in Table 3.

**Table 3:** The number of differential expressed transcripts and genes identified across the different intestinal region comparisons.

	Differential Expressed Transcripts	Differential Expressed Genes
Duodenum VS Cecum	2,593	2,151
Duodenum VS Colon	2,549	2,102
Cecum VS Colon	1,362	1,165
Total	4,221	3,254

To validate the RNA-seq data, the expression of 12 differentially expressed transcripts, including 5 randomly selected transcripts and 7 differently expressed highest expression transcripts, were further analyzed with quantitative real-time-PCR (qRT-PCR). As shown in Supplementary Table 2, the expression profiles of all 12 transcripts were consistent with those from RNA-seq.

However, since the differential expression analysis was applied to the two biological replicates separately, partial transcripts showed opposite expression pattern between two replicates. Moreover, the expression pattern of each transcript on three intestinal regions is still unknown. Therefore, to screen for the transcripts that meet our requirements, we performed hierarchical clustering on differentially expressed transcripts in subsequent analyzes.

### Functional enrichment analysis of differentially expressed transcripts between the duodenum and the large intestine

**Figure 1:**
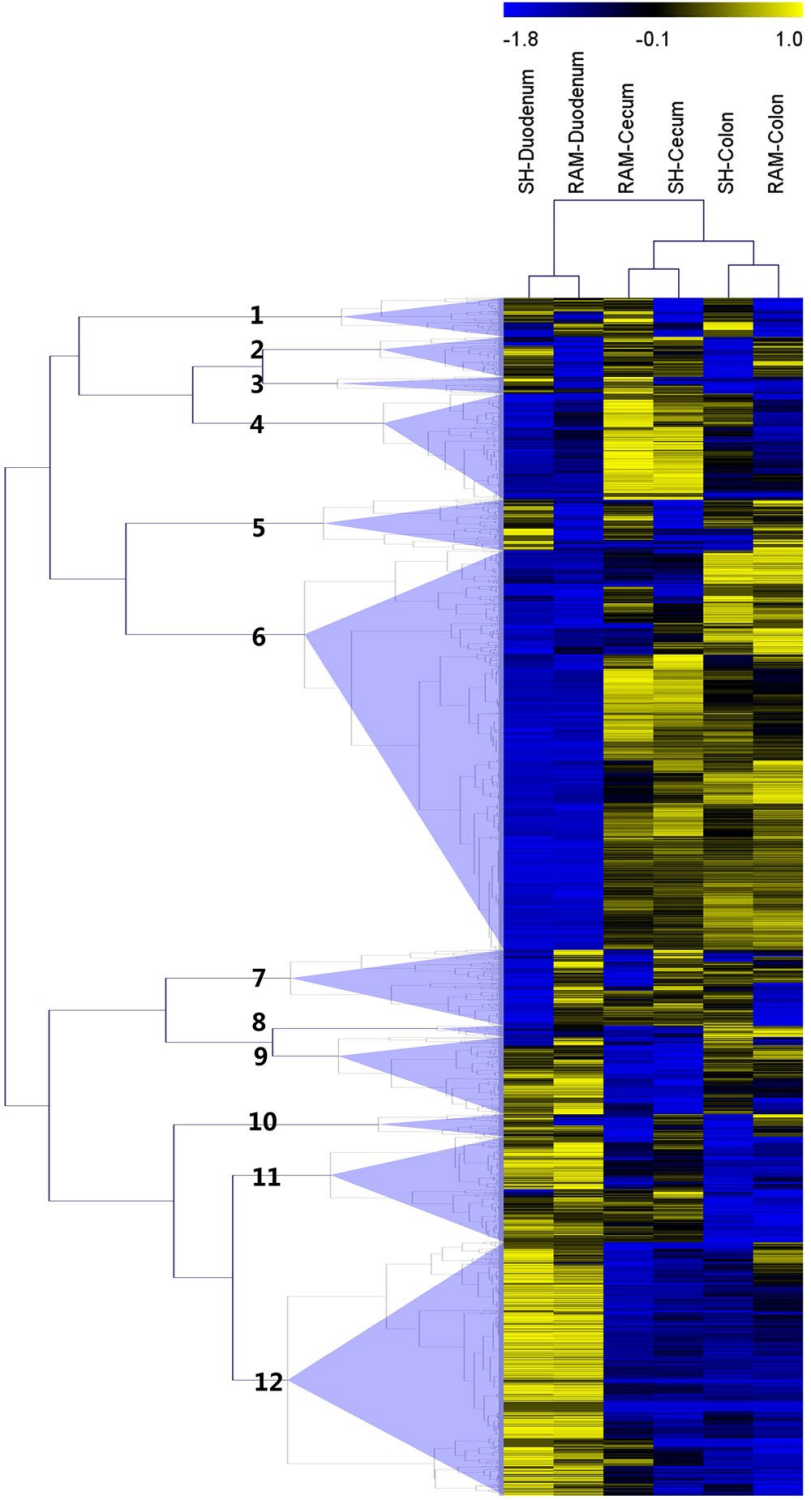
Clustered heat map showing the expression patterns of transcripts differentially expressed between the duodenum and the large intestine.

To further address the functions of transcripts that were differentially expressed between the duodenum and the large intestine, transcripts were clustered into 12 groups on the basis of their expression levels (Fig. 1). Many differentially expressed transcripts exhibited similar expression patterns. In groups 4, 8, and 12, transcripts were predominately higher expressed in one specific tissue (the colon, cecum and duodenum, respectively). In group 6, a large number of transcripts were higher expressed in both the cecum and colon. In group 9, a group of transcripts was higher expressed in the duodenum and colon, whereas the transcripts in group 11 were higher expressed in the duodenum and cecum. In groups 1, 2, 3, 5, 7 and 10, transcripts showed different expression patterns between SH and Texel. Given our goal of studying the difference between the duodenum and large intestine, groups 6 and 12 were used in further analyses.

On the basis of the expression profile cluster, differentially expressed transcripts were classified into different GO terms. As a result, the differentially expressed transcripts were associated with 157 GO terms, among which 6 terms were significantly enriched (Supplementary Dataset 2). Notably, two significantly enriched biological processes were associated with lipid metabolism: these processes were cholesterol homeostasis and fatty acid beta-oxidation.

To further understand the regulatory differences between the duodenum and large intestine, we mapped all transcripts from group 6 and 12 to KEGG pathways, with the goal of searching for transcripts involved in pathways that were significantly enriched. With a p-value ≤ 0.05 as the threshold, differentially expressed transcripts were found to be significantly enriched in 60 KEGG pathways (Supplementary Dataset 3). To examine whether these pathways were associated with highly expressed transcripts, we performed a pathway analysis on all medium and highly expressed transcripts (≥10 FPKM) of the co-enriched pathways. We found that 43 pathways achieved significant hits (Supplementary Dataset 3).

**Figure 2:**
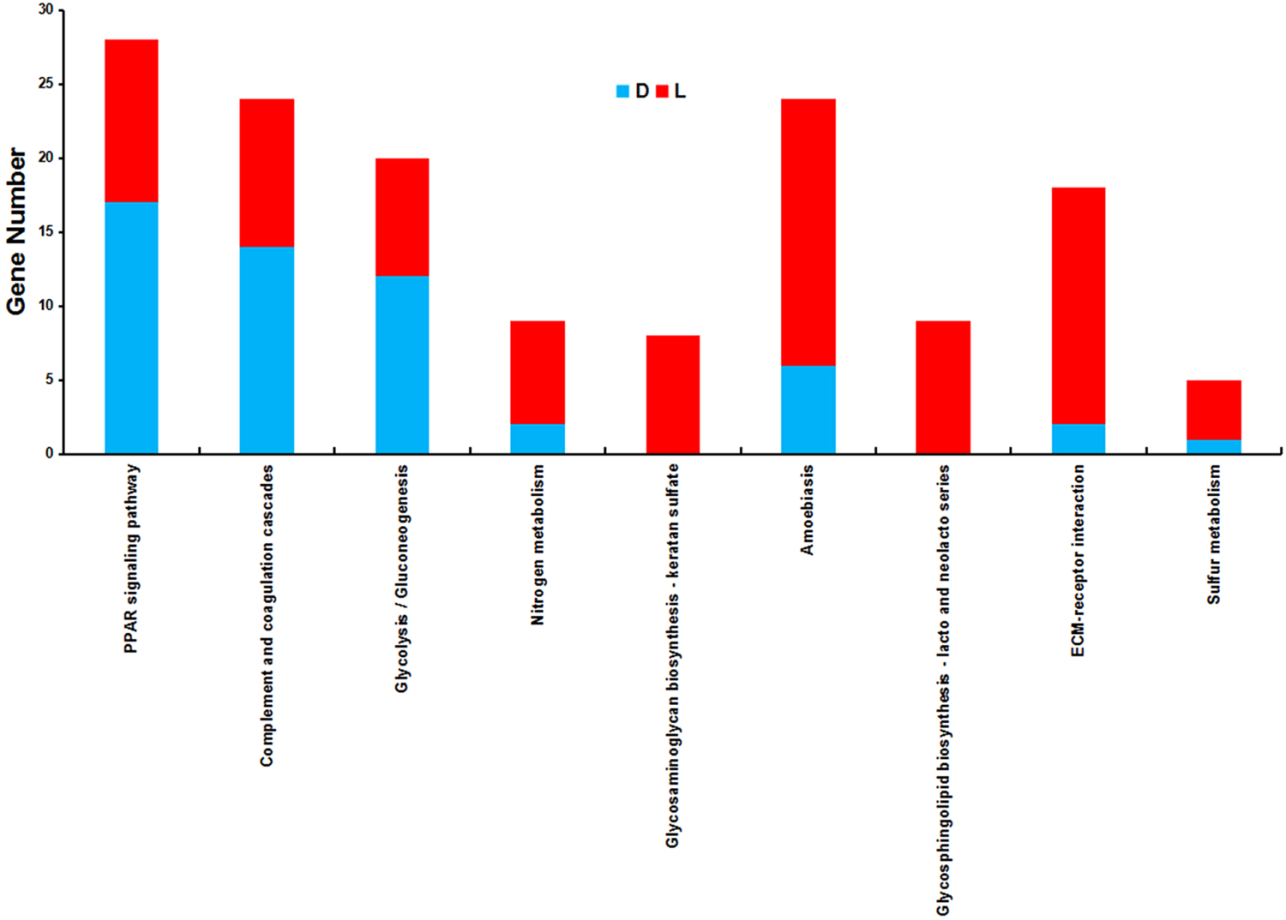
KEGG pathways achieved significant enrichment levels in all four enrichment analyzes. D: Duodenum higher expressed genes; L: Large intestine higher expressed genes.

Because our research focused on the large intestine, another KEGG pathway enrichment analysis was performed with transcripts that were higher expressed in the large intestine (group 6). As a result, 27 KEGG pathways were found to be significantly enriched (Supplementary Dataset 4), among which 14 achieved significant hits at the level of FPKM ≥ 10 (Supplementary Dataset 4). A total of 9 pathways achieved significant enrichment levels in all four KEGG pathway enrichment analyses (Fig. 2). Among them, 6 pathways were metabolism related, including the PPAR signaling pathway, nitrogen metabolism, glycosphingolipid biosynthesis - lacto and neolacto series, glycosaminoglycan biosynthesis - keratan sulfate, glycolysis/gluconeogenesis, and sulfur metabolism. Therefore, the 97 differentially expressed transcripts from the 6 pathways were subjected to further analysis. Performed with gene symbol annotation, the 97 differentially expressed transcripts were found to be encoded by 81 genes.

**Figure 3:**
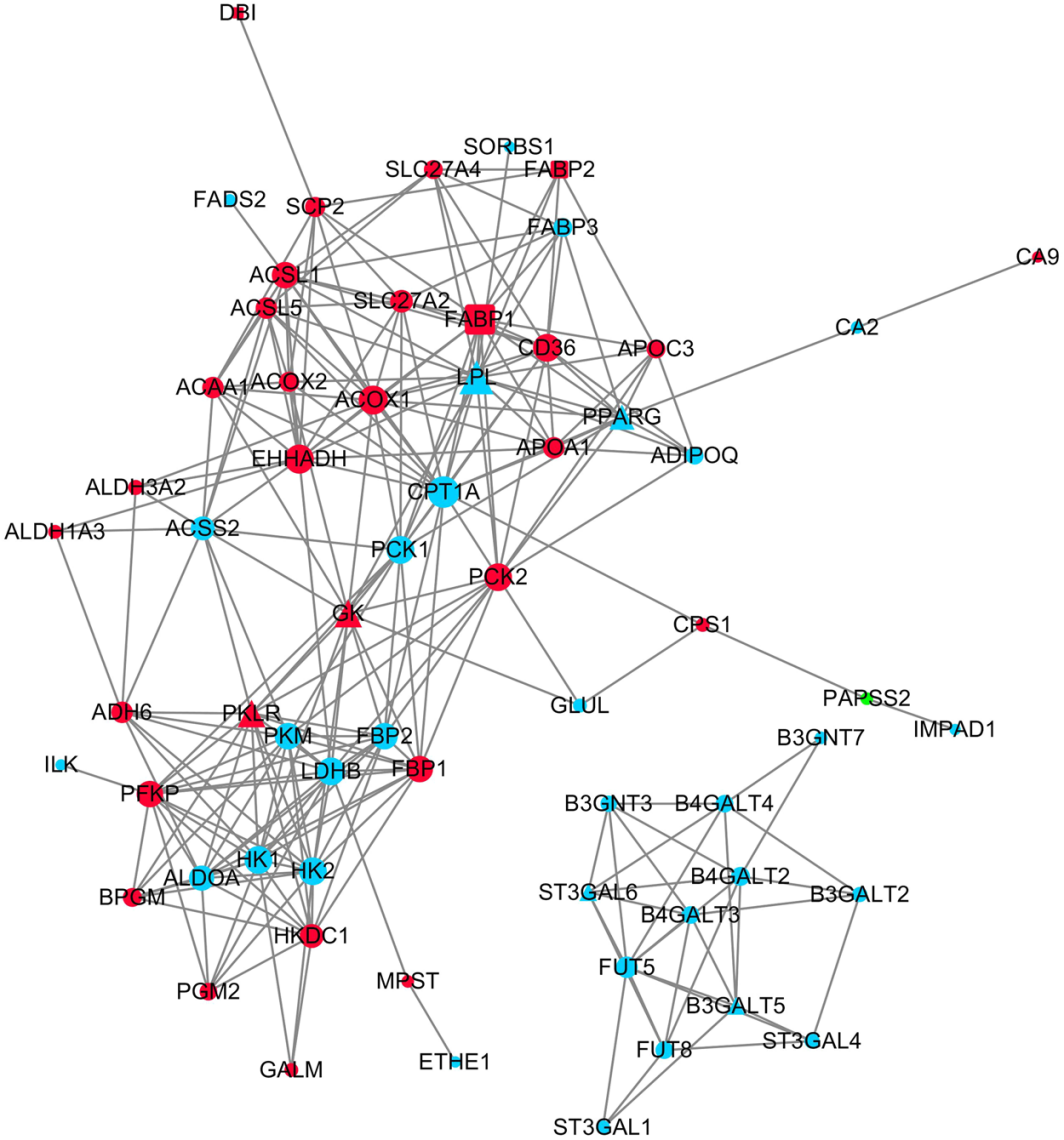
Protein-protein interaction networks of metabolism related pathway enriched genes. The node size was decided on the basis of the interaction degree value of each gene. Triangle node: FPKM < 10 in all intestinal regions; Circular node: 10 ≤ FPKM < 500 in at least one tissue with the highest expression; Square node: FPKM ≥ 500 in at least one intestinal tissue. Red: Higher expressed in the duodenum; Blue: Higher expressed in the large intestine; Green: Multiple transcript expression patterns are not consistent.

We performed a protein-protein interaction analysis of all 81 genes that were significantly enriched in the 6 metabolism related pathways. A total of 253 interactions between 63 genes were obtained from STRING (Fig. 3). Seven genes (*LPL*, *CPT1A*, *FABP1*, *EHHADH*, *ACOX1*, *CD36*, *PCK1*) were identified as hub genes (top 10% of interaction degree genes) of the network, whereas three genes were higher expressed in the large intestine (Lipoprotein lipase (*LPL*), Carnitine palmitoyltransferase 1A (*CPT1A*) and Phosphoenolpyruvate carboxykinase 1 (*PCK1*)).

### Functional enrichment analysis of differentially expressed genes between the cecum and colon

**Figure 4:**
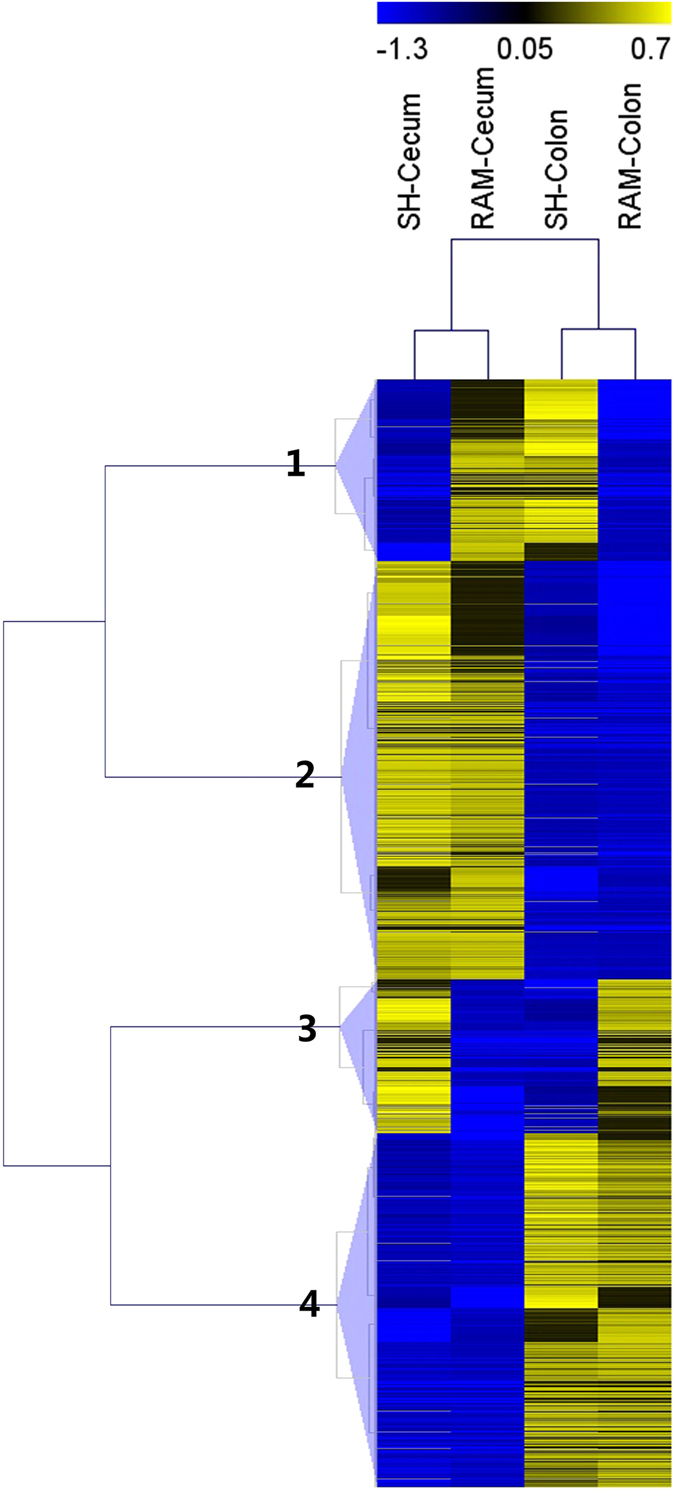
Clustered heat map showing the expression patterns of transcripts differentially expressed between the cecum and the colon.

To further study the differences in transcript expression between the cecum and colon, all 1,362 transcripts that were differentially expressed between the cecum and colon were clustered into 4 groups on the basis of their expression levels (Fig. 4). In group 1 and 3, transcripts showed different expression patterns between SH and Texel. In group 2 and 4, transcripts were predominately higher expressed in the cecum and the colon, respectively. Transcripts from group 2 and 4 were subjected to GO enrichment analysis and KEGG pathway enrichment analysis. In the GO enrichment analysis, differentially expressed genes were associated with 58 GO terms, among which no GO terms were significantly enriched.

**Figure 5:**
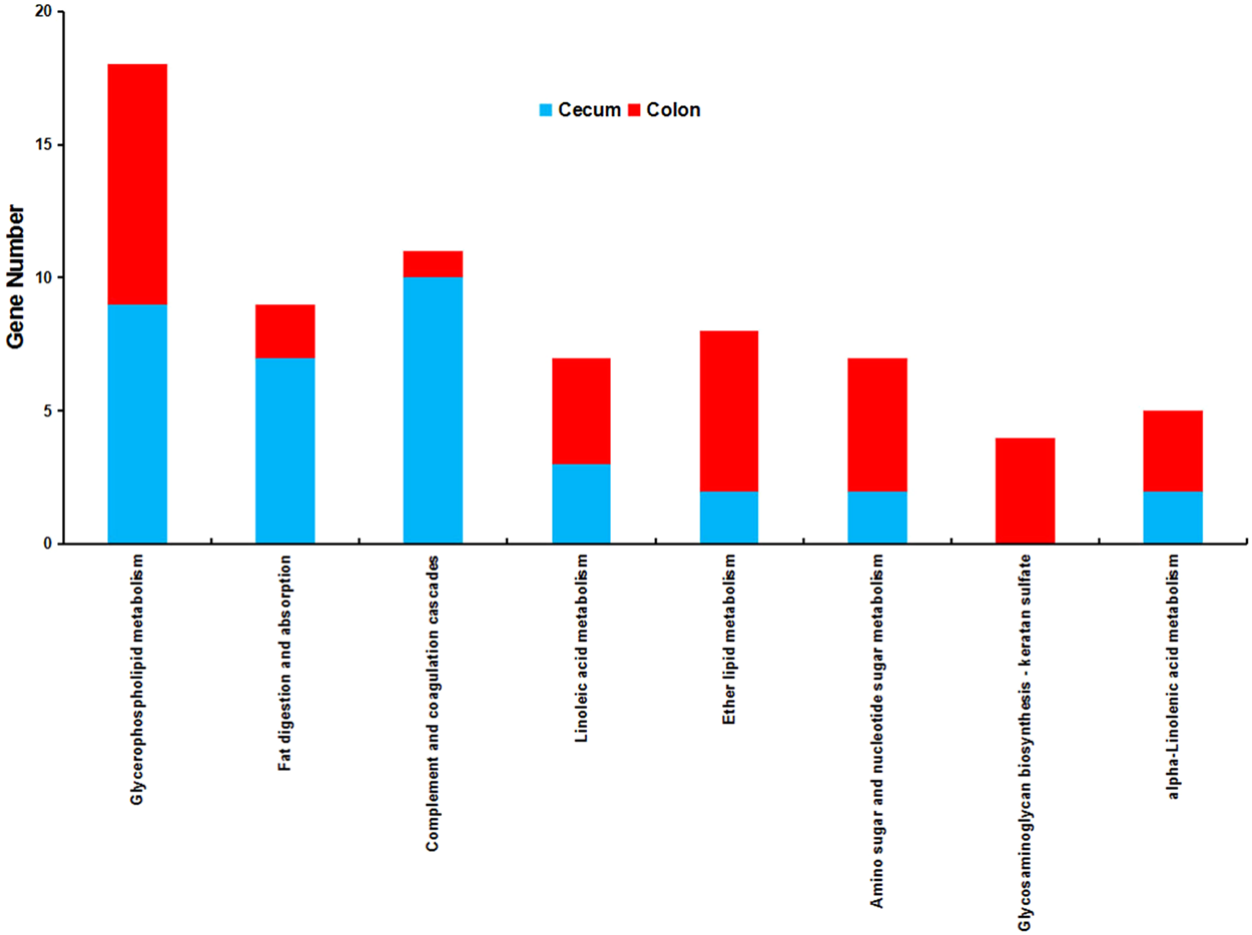
KEGG pathways achieved significant enrichment between the cecum and colon at both the FPKM ≥ 1 and FPKM ≥ 10 level. Cecum: Cecum higher expressed genes; Colon: Colon higher expressed genes.

In the KEGG pathway enrichment analysis, differentially expressed genes were significantly enriched in 16 pathways, among which 11 pathways were metabolism related (Supplementary Dataset 5). Re-performing the medium and highly expressed genes (≥10 FPKM in at least one intestine region) from significantly enriched pathways to the KEGG pathway enrichment indicated that only 8 pathways still reached a significant enrichment level (Fig. 5), whereas 5 pathways were lipid metabolism related (Supplementary Dataset 5). These included the glycerophospholipid metabolism, fat digestion and absorption, linoleic acid metabolism, ether lipid metabolism, and alpha-Linolenic acid metabolism pathways. This finding suggests that the lipid metabolism associated pathways were detected because of generally moderate and high levels of gene expression.

**Figure 6:**
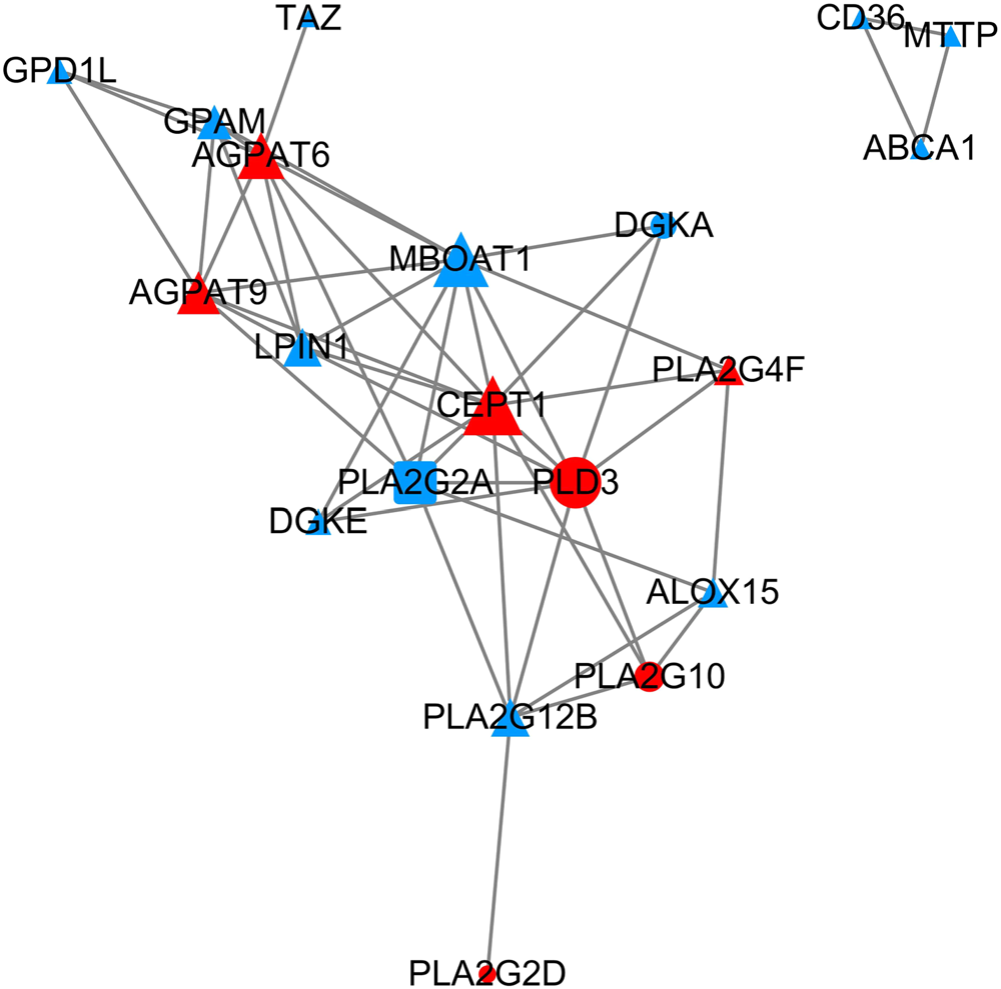
Protein-protein interaction networks of lipid metabolism related pathway enriched genes. The node size was decided on the basis of the interaction degree value of each gene. Triangle node: FPKM < 10 in either the cecum or colon; Circular node: 10 ≤ FPKM < 500 in the tissue with the highest expression; Square node: FPKM ≥ 500 in at least one intestinal tissue. Red: Higher expressed in colon; Blue: Higher expressed in cecum; Green: Multiple transcript expression patterns are not consistent.

A total of 28 transcripts were significantly enriched in these 5 lipid metabolism-related pathways, among which 24 genes were constructed. Among them, 14 genes were higher expressed in the cecum and 10 genes were high expressed in the colon. All 24 genes were used for further protein-protein interaction analysis. As a result, a total of 49 interactions between 20 genes were obtained (Fig. 6). Two hub genes were identified with interaction degree ranking (top 10% interaction degree genes): Choline/Ethanolamine phosphotransferase 1 (*CEPT1*) and the Membrane bound O-acyltransferase domain containing 1 (*MBOAT1*).

## Discussion

For ruminants, the rumen and small intestine are important organs for fat absorption and triacylglycerol homeostasis. However, what is often overlooked is the important role played by the large intestine in fat absorption. The primary chambers of the rumen facilitate the action of a complex mixture of micro-organisms in fermenting a portion of the plant polysaccharides and lipids to short-chain volatile fatty acids (SCFAs), principally acetate, butyrate and propionate^26^. SCFAs are the primary carbon energy source of ruminants, and the rumen is the major site of their uptake^26^. As the secondary fermentation organ, the large intestine accounts for 8 to 17% of total volatile fatty acids absorbed from the digestive tract, mostly SCFAs^4, 8^. However, the overlap in functions of the rumen and the large intestine created inconsistencies at the molecular regulation level. Xiang *et al*. reported that compared to the large intestine, the rumen was more metabolically similar to the liver, especially for lipid metabolism^25^. Therefore, to improve the understanding of sheep large intestinal metabolism, detection of lipid metabolism should be a primary objective. The small intestine mainly absorbs long and medium chain fatty acids (LCFAs and MCFAs) that escaped fermentation in the rumen^1^. Based on their functional differences, a differential expression analysis between the large intestine and small intestine will help elucidate the characteristics of large intestine metabolism.

In sheep, as in other herbivores, the ruminant large intestine plays a crucial role in digestion and absorption. To understand the complex developmental and regulation processes, and to expose molecular events within cells and tissues, whole transcriptome analysis is necessary^27^. In this study, we performed transcriptome analysis for intestinal mucosal epithelial tissues collected from sheep cecum, colon and duodenum. To ensure the accuracy of analysis results, an FPKM score of 1.0 in at least one sample was chosen as a minimum expression cutoff for transcripts, and different levels of expression cutoffs were used in KEGG enrichment analysis. It has been shown in several studies that the power to detect differential expressed transcript units improves when the number of biological replicates increases^28–30^. This phenomenon is interpreted as a result of an increase in detection sensitivity, especially for low expression transcripts^30^. Furthermore, as n (the number of biological replicates) increases, both algorithms increase the call rate and true positive rate (TPR) while the false positive rate (FPR) remains unchanged^28^. Therefore, we believe that the exclusion of low expression genes in our study (with only 2 biological replicates) can improve the reliability of differential analysis results. However, what must be emphasized is the statistical power has been reduced since the data comes from two different experiments. Moreover, the unconventional method we used to remove batch effect also made it impossible to calculate the statistical power accurately. It can be deduced that the statistical power of this study is lower than those with at least 2 biological replicates, but higher than that of non-biological replicate studies. Therefore, the statistical power of our design is relatively limited, but should still be effective to describe the individual variability that exists in sheep.

A total of 3,254 genes encoding 4,221 transcripts were identified as differentially expressed. The aim of this study was to assess the transcript expression differences between the large intestine and the duodenum of the sheep, and between the cecum and colon in the sheep large intestine, thereby possibly contributing to an improved understanding of regulation of sheep intestinal metabolism.

Of 60 KEGG pathways enriched in the differentially expressed transcripts between the duodenum and the large intestine, more than half were metabolism related. However, KEGG pathway analysis of the transcripts that had higher expression in the large intestine revealed that only six metabolic pathways were significantly enriched in both analyses at moderate or high expression levels. Among the six pathways mentioned above, both carbohydrate and lipid metabolism pathways were observed.

All 7 hub genes identified to be differentially expressed between the duodenum and large intestine were enriched in the PPAR signaling pathway, whereas 3 large intestine highly expressed hub genes were identified. These results indicated that the PPAR signaling pathway may play a central regulatory role in intestinal metabolism. PPAR signaling is mainly associated with metabolism, cell development, adipocyte differentiation and cell survival^31–34^. In the duodenum, 17 genes were significantly enriched in the PPAR signaling pathway, and 11 were significantly enriched in the large intestine. Among the three PPARs, only *PPARG* showed higher expression in the cecum and colon. Such results were consistent with the previously reported PPAR tissue expression specificity^34–36^.

Recently, two putative pathways for uptake of MCFAs and LCFAs have been identified^37^: CD36, alone or together with the FABP1, accepts MCFAs and LCFAs at the cell surface. Once at the inner side of the membrane, these fatty acids are bound by FABP3 before entering metabolic pathways. MCFAs and LCFAs can also be transported by SLC27A4, after which these fatty acids could then be activated by ACS1 to form acyl-CoA esters^37^. In our study, all MCFAs and LCFAs absorption essential genes (*CD36*, *FABP1*, *SLC27A4* and *ACS1L*), except *FABP3*, had higher expression in the duodenum. In addition, the major cholesterol absorption essential genes (*NPC1L1, ABCG5* and *ABCG8*) and chylomicrons forming essential genes (*ACAT2*, *APOB* and *MTTP*) were also expressed more in the duodenum. This result is consistent with our expectation that the duodenum mainly absorbs MCFAs and LCFAs. It is worth noting that the major fatty acid beta-oxidation related genes are also highly expressed in the duodenum, which is similar to the result reported by Xiang *et al*., in the rumen, duodenum, cecum and colon^25^. This result may suggest that the use of fatty acid as a directive energy source in the duodenum is more favorable compared to the large intestine.

On the other hand, there is a group of SCFA metabolism-related genes that showed higher expression in the large intestine than in the duodenum. Although SCFAs can pass through the top membrane by simple passive diffusion and be absorbed into the portal vein, carrier-mediated transport still plays an important role in the uptake of SCFAs^38^. In this study, two potent SCFA transporters, *SLC5A8* (also known as sodium-coupled monocarboxylate transporter 1, *SMCT1*) and *SLC16A1* (also known as monocarboxylate transporter 1, *MCT1*) were expressed more in the large intestine, which could show that SCFA absorption largely occurs in the large intestine. Studies in various species showed that SCFAs are efficient gluconeogenic substrates^39, 40^ and crucial energy substrates for enterocytes^41^. Consistently, a number of glycolysis/gluconeogenesis pathway genes were found to have higher expression in the large intestine than in the duodenum.

Three more highly expressed hub genes in the large intestine were identified by PPI network analysis between the duodenum and the large intestine, of which all three genes were enriched in PPAR signaling pathway. Among them, *PCK1* encodes a cytosolic enzyme that, along with GTP, catalyzes the formation of phosphoenolpyruvate from oxaloacetate^42^. Regulated by *PPARG*, *PCK1* also controls glyceroneogenesis and lipid production^43^. Showing higher expression levels in the large intestine, *PCK1* may play an important regulatory role in glycolysis/gluconeogenesis of the large intestine. However, the higher expression of another two hub genes (*LPL* and *CPT1A*) seems to be somewhat contradictory to the higher expression level of *PCK1*.

As a rate-limiting enzyme, LPL has been reported to be involved in promoting the production of chylomicron remnants, cholesterol-rich lipoproteins, and free fatty acid uptake^44, 45^. Overexpression of *LPL* has been shown to affect insulin response and promote obesity^46^. By encoding a key lipolytic enzyme, carnitine palmitoyltransferase 1A, the *CPT1A* gene controls the entrance of fatty acids into the mitochondria for beta-oxidation^47^. In addition, we also noticed that lipid metabolism-related PPAR signaling pathway genes *FABP3*, *FADS2*, *LOC101108497*, *ADIPOQ* and *SORBS1* were highly expressed in the large intestine. To confirm whether this result is related to lipogenesis and adipocyte differentiation, we examined the expression values of major adipocyte lipogenesis genes (*ACACA*, *FASN* and *SCD*) and TAG synthesis and storage pathway genes (*ACSM1*, *GPAM*, *AGPAT2*, *LPIN1*, *DGAT2*, *CIDEC*, *CIDEA*, *FABP4* and *PLIN1*). As a result, we found that major adipocyte lipogenesis genes (*ACACA*, *FASN* and *SCD*) showed similar expression levels between the duodenum and large intestine. Genes *ACSM1*, *DGAT2*, *CIDEC*, *CIDEA*, *FABP4* and *PLIN1* did not achieve significant differential expression levels between intestinal regions. Genes *GPAM* and *LPIN1* showed higher expression in the duodenum. Only gene *AGPAT2* showed higher expression in the large intestine region. This result precludes the possibility that adipocytes are mixed into large intestine samples. Such results also revealed the presence of unique lipid metabolism in the sheep large intestine. However, due to the lack of evidence on a metabolomics level, it is difficult to make a more detailed hypothesis for the specific mechanism of sheep large intestine.

Of the 16 KEGG pathways significantly enriched with differentially expressed transcripts between the cecum and colon, only 8 remained at the level of FPKM ≥ 10. This result suggests that most of these enriched pathways were detected due to low levels of gene expression. In previous reports, the colon has been confirmed to be weaker than the cecum in fermentation and absorption functions^3^. Correspondingly, 7 of the 9 fat digestion and absorption pathway genes were highly expressed in the cecum, which could be a manifestation of their differences in lipid absorption capacity.

The two hub genes detected between the cecum and the colon, *MBOAT1* showed higher expression in the cecum, while *CEPT1* was found to be higher expressed in the colon. *CEPT1* encodes a choline/ethanolamine phosphotransferase, which functions in the synthesis of choline and ethanolamine containing phospholipids^48^. The knockdown of *CEPT1* in the liver suppresses the expression of PPARA-dependent genes^49^. As a member of the membrane bound O-acyl transferase family^50^, *MBOAT1* is a lysophosphatidylserine acyltransferase with a preference for oleoyl-CoA^51^. MBOAT1 has been reported to be associated with neuronal development^52^ and deregulation of phosphatidylcholine metabolism^53^. The function of the *MBOAT1* gene in the large intestine may be related to apoptosis and modulation of the activities of transporters and membrane-bound enzymes. More research on these genes will be necessary to uncover their specific functions in lipid metabolism in the large intestine.

Overall, in this study, we observed the differential expression of metabolism-related genes among the duodenum, cecum and colon. On the basis of expression pattern clustering, GO enrichment, KEGG enrichment and PPI network analysis, a group of metabolic related genes and pathways were found to be regionally enriched in different intestinal segments. Our findings suggest that the PPAR signaling may be a key driver of metabolic differences between the large intestine and the duodenum in sheep. Large intestine higher expressed hub genes *CPT1A*, *LPL* and *PCK1* may play important regulatory roles in intestinal metabolism through the PPAR signaling pathway. This study also reveals that differences in lipid metabolism between sheep cecum and colon may be due to differential gene expression in Glycerophospholipid metabolism, Fat digestion and absorption, Linoleic acid metabolism, Ether lipid metabolism, and the alpha-Linolenic acid metabolism pathway. Two hub genes, *CEPT1* and *MBOAT1*, may play important roles in the lipid metabolism regulation in the large intestine.

## Materials and Methods

### Ethics Statement

All animal experiments were approved by the Institutional Animal Care and Use Ethics Committee of Shandong Agricultural University (Permit Number:2004006) and were performed in accordance with the “Guidelines for Experimental Animals” of the Ministry of Science and Technology (Beijing, China). All surgery was performed according to recommendations proposed by the European Commission (1997), and all efforts were made to minimize suffering.

### Animals and Sampling

Duodenum, cecum and colon tissue samples were collected from three healthy 11-month-old small-tailed Han ewes (Linqv Huanong Sheep Farm, Weifang, Shandong, China). The appearance and shape of the sheep was fully consistent with their varietal characteristics. The selected sheep were healthy with moderate weight. All the sheep were raised under the same conditions, which included natural lighting and free access to water and food. The intestine tissues were collected immediately after exsanguination. Duodenal tissue and cecal tissues were sampled from the middle section of each intestinal region. The colon tissue was sampled from the middle section of the proximal colon. Mucosal epithelial tissues from three intestinal regions were placed into liquid nitrogen and stored at −80 °C.

### RNA Extraction and Sequencing

The total RNA of mucosal epithelial tissues from each sample was extracted using the TRIzol reagent according to the manufacturer’s protocol (Invitrogen, Burlington, ON, Canada). DNA was removed from each sample by using RNase-free DNase (New England Biolabs). The RNA integrity and concentration was evaluated with an Agilent Technologies 2100 Bioanalyzer. Both samples had RNA Integrity Number (RIN) values greater than 7.5.

The transcriptome secquencing library construction and Illumina sequencing were performed at the Beijing Genomics Institute (Shenzhen, China), according to the manufacturer’s instructions (Illumina, San Diego, CA). The fragments were sequenced on an Illumina HiSeq 2000 platform, thus generating paired-end reads of 100 bp. The deep sequencing data obtained have been deposited in the GEO database with the accession number GSE92656.

### Mapping, Annotation and Identification of Differentially Expressed Transcripts

Before transcriptome assembly, we carried out a filtering process of SH raw sequencing reads. Reads with adaptor contamination, more than 50% of bases with a quality score of Q < 10, or more than 10% ambiguous sequences represented as “N” were removed. Clean Texel read files were downloaded from the Ensembl database (ENA study accession PRJEB6169)^24^.

Using Bowtie2^54^ and RSEM^55^, we mapped and assembled our reads with the sheep reference genome v4.0 (ftp://ftp.ncbi.nlm.nih.gov/genomes/Ovis_aries/) and reference annotated transcripts (ftp://ftp.ncbi.nlm.nih.gov/genomes/all/GCF/000/298/735/GCF_000298735.2_Oar_v4.0/GCF_000298735.2_Oar_v4.0_rna.fna.gz).

The detection of transcript expression levels was performed by RSEM^55^, on the basis of the number of fragments per kilobase of exon per million fragments mapped (FPKM) of the transcripts. The principal components analysis was performed with SPSS (Version 19.0, IBM, USA). Transcript expression value mean center was performed with z-value transformation.

Differentially expressed transcripts and corresponding p-values were determined using methods described by Audic and Claverie^56^, and the false discovery rate (FDR) was calculated on the basis of Benjamini and Hochberg multiple testing correction^57^. The significantly differentially expressed transcripts were declared at a fold change ≥ 2 and a false discovery rate (FDR) < 0.001. Using this method, the differentially expressed transcripts were identified among the duodenum, cecum and colon, through a pairwise comparison analysis.

### Clustering heat map

The expression of differentially expressed transcripts was gene mean centered and visualized a heat map using the MultiExperiment Viewer (MeV)^58^. Where indicated on the dendrograms, Pearson correlation average linkage hierarchical clustering was performed.

### GO term enrichment analysis and KEGG Pathway analysis

After expression pattern clustering, the transcripts from specific groups were subjected to functional annotation, including GO (Gene Ontology) functional annotation and KEGG (Kyoto Encyclopedia of Genes and Genomes) pathway annotation. The GO terms and KEGG pathway enrichment was performed using The Database for Annotation, Visualization and Integrated Discovery (DAVID v 6.8, http://david.abcc.ncifcrf.gov)^59, 60^.

### Protein-protein interaction networks

PPI (Protein-protein interaction) networks were constructed on the basis of information from STRING v10.0, which provides critical assessment and integration of protein-protein interactions, including direct (physical) and indirect (functional) associations^61^. Credible interactions (combined_score ≥ 0.4) were accepted for further network analysis using CytoScape^62^. Network module analysis and hub gene identification were subjected to the MCODE method^63^ by using the plugin MCODE of CytoScape. The node size was determined to be proportional to the interaction degree.

### Quantitative real-time PCR validation

The qRT-PCR primers used for validation were designed according to the sequences of selected identified transcripts by using Primer Premier 5.0 (Premier, Canada). Primer sequences are shown in Additional file 1. The cDNA was synthesized by using Primerscript RT reagent Kit (TaKaRa), with 1 μg of total RNA. qRT-PCR was performed using SYBR Premix Ex Taq II (TaKaRa, DRR081 A), 7.5 mL of 2X SYBR Premix Ex Taq II reaction buffer, 0.3 mL of primers (0.4 mmol each), 0.3 mL of 50X ROX Reference Dye II, 1.5 mL of cDNA (10 pg/mL,1 mg/mL), and RNase-free dH_2_O to a final volume of 15 mL. The reaction mixtures were incubated in a 96-well plate in an Mx3000p SYBR Green real-time quantitative PCR analyzer (Stratagene, USA) at 95 µC for 30 sec, and this was followed by 40 cycles of 95 µC for 5 sec and 60 µC for 30 sec. All reactions were performed in triplicate. After amplification, melting curve analysis was performed to verify the specificity of the reactions using the Mx3000/Mx Pro software (Stratagene, USA). The primers of the housekeeping gene *ACTB* used for the loading control are described in Supplementary Table 1.

## Electronic supplementary material

Supplementary Information

Dataset 1

Dataset 2

Dataset 3

Dataset 4

Dataset 5
