# Supplementary material for: Transcriptome Analysis of Three Sheep Intestinal Regions reveals Key Pathways and Hub Regulatory Genes of Large Intestinal Lipid Metabolism
Source: Sci Rep. 2017 Jul 13;7:5345. doi: 10.1038/s41598-017-05551-2 (PMC5509726; doi:10.1038/s41598-017-05551-2)
Supplement: Supplementary file 1 — Supplementary Information [file 41598_2017_5551_MOESM1_ESM.pdf]

Transcriptome Analysis of Three Sheep Intestinal Regions reveals Key Pathways and Hub Regulatory Genes of Large Intestinal Lipid Metabolism

Tianle Chao[1], Guizhi Wang, Zhibin Ji, Zhaohua Liu, Lei Hou, Jin Wang, Jianmin Wang[1]*

[1] Shandong Provincial Key Laboratory of Animal Biotechnology and Disease Control and Prevention, College of Animal Science and Veterinary Medicine, Shandong Agricultural University, Taian 271018, China

*Corresponding author

E-mail: wangjm@sdau.edu.cn(JMW)

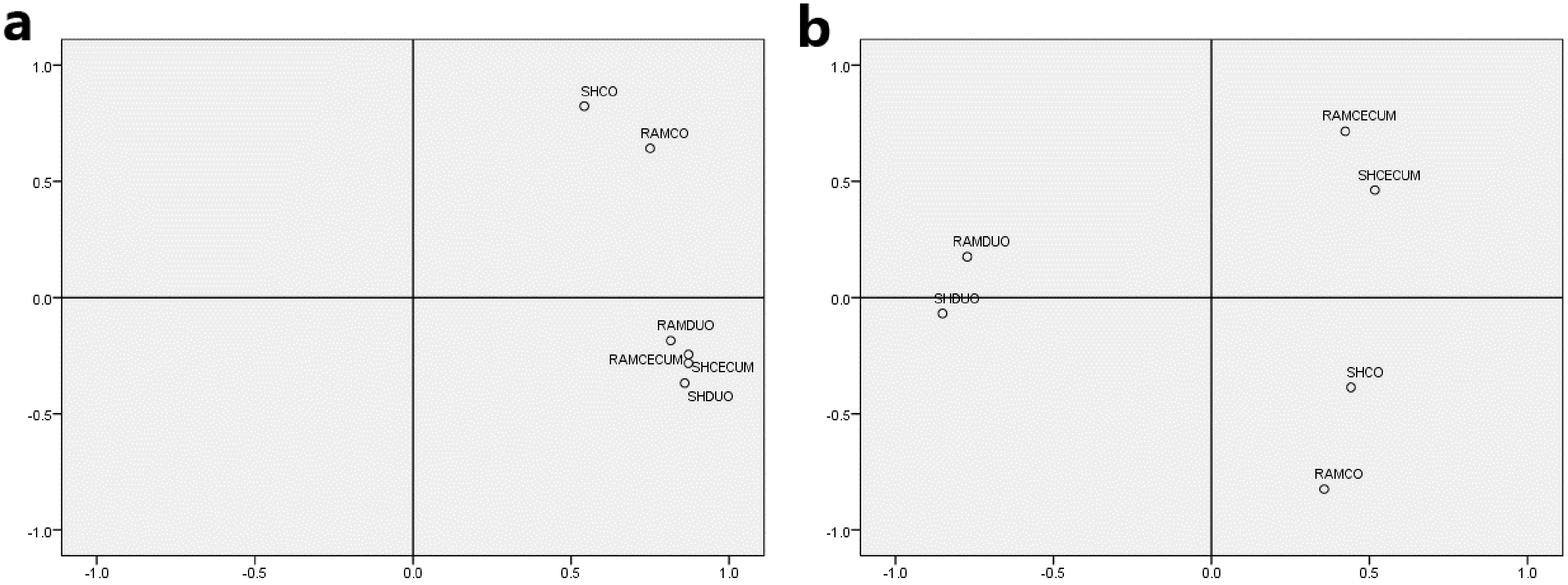

Supplementary Figure 1. Principal component plot of transcripts expression data. RAMDUO: Texel ram duodenum. RAMCECUM: Texel ram cecum. RAMCO: Texel ram colon. SHDUO: Small-tailed Han duodenum. SHCECUM: Small-tailed Han cecum. SHCO: Small-tailed Han colon. **(a)** Principal component plot of original expression data. **(b)** Principal component plot of mean centered expression data.

Supplementary Table 1. Primers for quantitative real-time PCR validation

Gene	Transcript ID	Forward primer	Reverse primer
TFF2	XM_004003362.2	CACTTGGCTCCTGGCTACGC	GCCGCAGTTCACCCTGTTGT
SPINK4	XM_004004147.3	CTGGCCTTGGCTGTTCTCTT	GCTTGCATTCACTCTCATACGTG
REG4	XM_004002391.2	CAGCAAAGAATGCAACGAGC	GCAGAGAAGGAAGAAGAAAGGG
LOC101103222	XM_004006473.3	ATGGCAAAACCCCAAGAGC	GACAGATGAGCAGAAGAAAGAAAAC
FABP1	XM_004005898.3	CAAATACCAAGTCCAGACCCAG	ATCTCACACTCCTCCCCCAA
LOC654331	NM_001038014.1	GCTCATCATGGACTTGGAACC	GGGGAGGAGGGAAAGACAGA
JCHAIN	XM_004009888.3	CAACAAATGTAAGTGTGCCCG	AGGTGTAACAGGTCTCAGTGTCTCT
ST6GALNAC6	XM_012162335.1	GTGATTGCGGTGGAGTTGTGT	GCTGTTCTCGTTCTGGATGTAGGT
LDHB	XM_004006792.3	GACTCAAGCGTGGCTGTGTG	AGGCACTCTCCACCACCATC
ADA	XM_015099501.1	CGTGGACACACTCAAGACCGA	CGAACAACTGGGTGCTCCG
ASS1	XM_004005582.2	CATCTCCCAAGTGTAGCTGCTAATT	CGCCCCATCCCTCTGTCT
LOC101107420	XM_012153432.1	AGAGGAACCCCCAAGGACG	CATAAGTGGCGTTGTATTTCAGGA
ACTB	n/a	CAAAGACCTCTACGCCAACACG	GGAGCCGCCAATCCACAC

Supplementary Table 2. Quantitative real-time PCR validation results.

Transcript ID	Gene Symbol	Duodenum mean ± SD	Cecum mean ± SD	Colon mean ± SD	Duodenum RNA-seq FPKM	Cecum RNA-seq FPKM	Colon RNA-seq FPKM
XM_004003362.2	TFF2	44.473 ± 18.224	45.577 ± 12.642	2393.759 ± 232.720	1301	1843.89	116052
XM_004004147.3	SPINK4	37.436 ± 9.149	106.368 ± 20.835	415.530 ± 73.649	589.11	3434.29	15164.92
XM_004002391.2	REG4	11.561 ± 2.773	37.282 ± 7.042	243.029 ± 31.243	256.53	1753.65	10367.78
XM_004006473.3	LOC101103222	37.566 ± 10.828	184.068 ± 59.667	89.287 ± 34.166	748.05	5724.33	1315.39
XM_004005898.3	FABP1	286.958 ± 88.195	5.753 ± 0.958	5.751 ± 0.687	6710.91	835.65	369.72
NM_001038014.1	LOC654331	76.387 ± 7.724	5.127 ± 1.389	0.045 ± 0.007	3184.85	444.17	3.42
XM_004009888.3	JCHAIN	330.653 ± 24.637	124.728 ± 7.088	24.690 ± 7.914	6791.44	3542.58	675.07
XM_012162335.1	ST6GALNAC6	0.060 ± 0.003	0.808 ± 0.139	2.138 ± 0.157	4.16	98.29	142.02
XM_004006792.3	LDHB	8.085 ± 1.539	17.849 ± 2.744	18.440 ± 1.615	76.94	388.87	352.11
XM_015099501.1	ADA	11.465 ± 2.187	0.155 ± 0.010	0.347 ± 0.012	571.62	19.22	22.36
XM_004005582.2	ASS1	0.197 ± 0.044	0.313 ± 0.042	1.456 ± 0.099	24.29	101.35	306.95
XM_012153432.1	LOC101107420	66.698 ± 18.560	2.944 ± 0.721	0.919 ± 0.073	2084.85	287.61	63.79
